# Supplementary material for: Cell cycle networks link gene expression dysregulation, mutation, and brain maldevelopment in autistic toddlers
Source: Mol Syst Biol. 2015 Dec 14;11(12):841. doi: 10.15252/msb.20156108 (PMC4704485; doi:10.15252/msb.20156108)
Supplement: Supplementary file 1 — Appendix [file MSB-11-841-s001.pdf]

APPENDIX

- TABLE OF CONTENT -

FIGURES

Appendix Figure S1. WGCNA combined analysis across ASD and control toddlers pg. 2
Appendix Figure S2. Module preservation analysis and quality scores pg. 3
Appendix Figure S3. Module preservation analysis between ASD and control datasets pg. 4
Appendix Figure S4. Permutation analysis of the seven modules associated with TBV measures in the combined WGCNA analysis pg. 5
Appendix Figure S5. Permutation analysis of the top modules associated with TBV measures in ASD and Control groups pg. 6
Appendix Figure S6. Association analysis between MEs and TBV measures using WGCNA on control toddlers pg. 7
Appendix Figure S7. Association analysis between MEs and TBV measures using WGCNA on ASD toddlers pg. 8
Appendix Figure S8. Boxplots and T-test analysis of Gene Significance pg. 9
Appendix Figure S9. Linear correlations for peripheral genes with high relevance to ASD brain measures pg. 10
Appendix Figure S10. Topological mapping of cell cycle hub-genes in a protein-protein interaction (PPI) network pg. 11

TABLES

Appendix Table S1. Module preservation analysis between ASD and control datasets pg. 12
Appendix Table S2. Bootstrapped (10,000 resamples) 95% confidence intervals for correlation estimates in TD and ASD pg. 13
Appendix Table S3. Linear regression analysis with age-correction and ANOVA testing pg. 13
Appendix Table S4. Permutation analysis testing between-group difference (TD vs ASD) in correlation strength (10,000 permutations) pg. 14
Appendix Table S5. Module preservation analysis between blood and BrainSpan datasets pg. 14
Appendix Table S6. Permutation analyses testing between-group difference (TD vs ASD) in correlation strength (10,000 permutations) pg. 15
Appendix Table S7. CHD8 subnetwork linear regression analysis with age-correction and ANOVA testing pg. 15

APPENDIX FIGURES

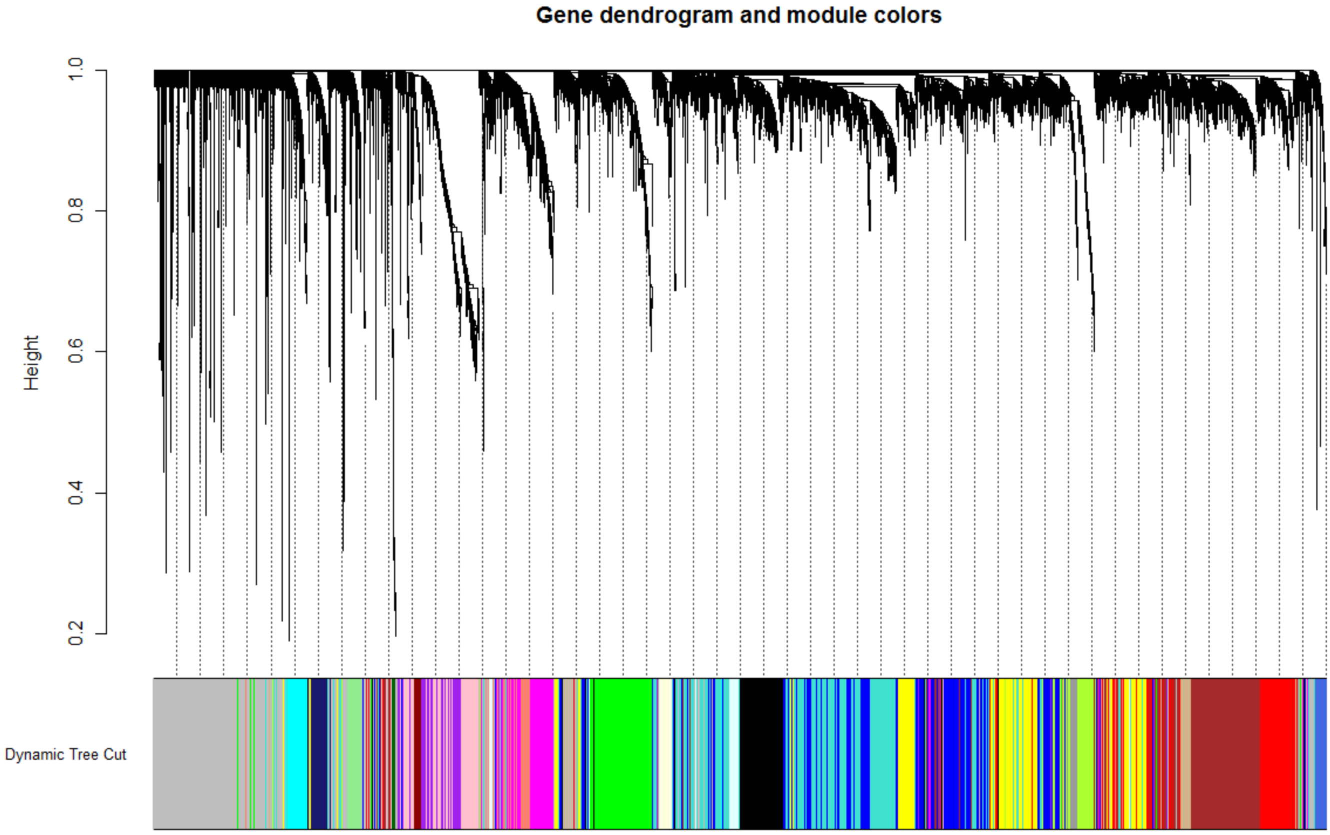

Appendix Figure S1. WGCNA combined analysis across ASD and control toddlers. Co-expression modules are generated and color-coded. Each vertical line corresponds to a gene, and genes with similar expression are clustered into modules. Modules are herein called by the assigned WGCNA default colors. Module eigengenes are computed for each subject and each module.

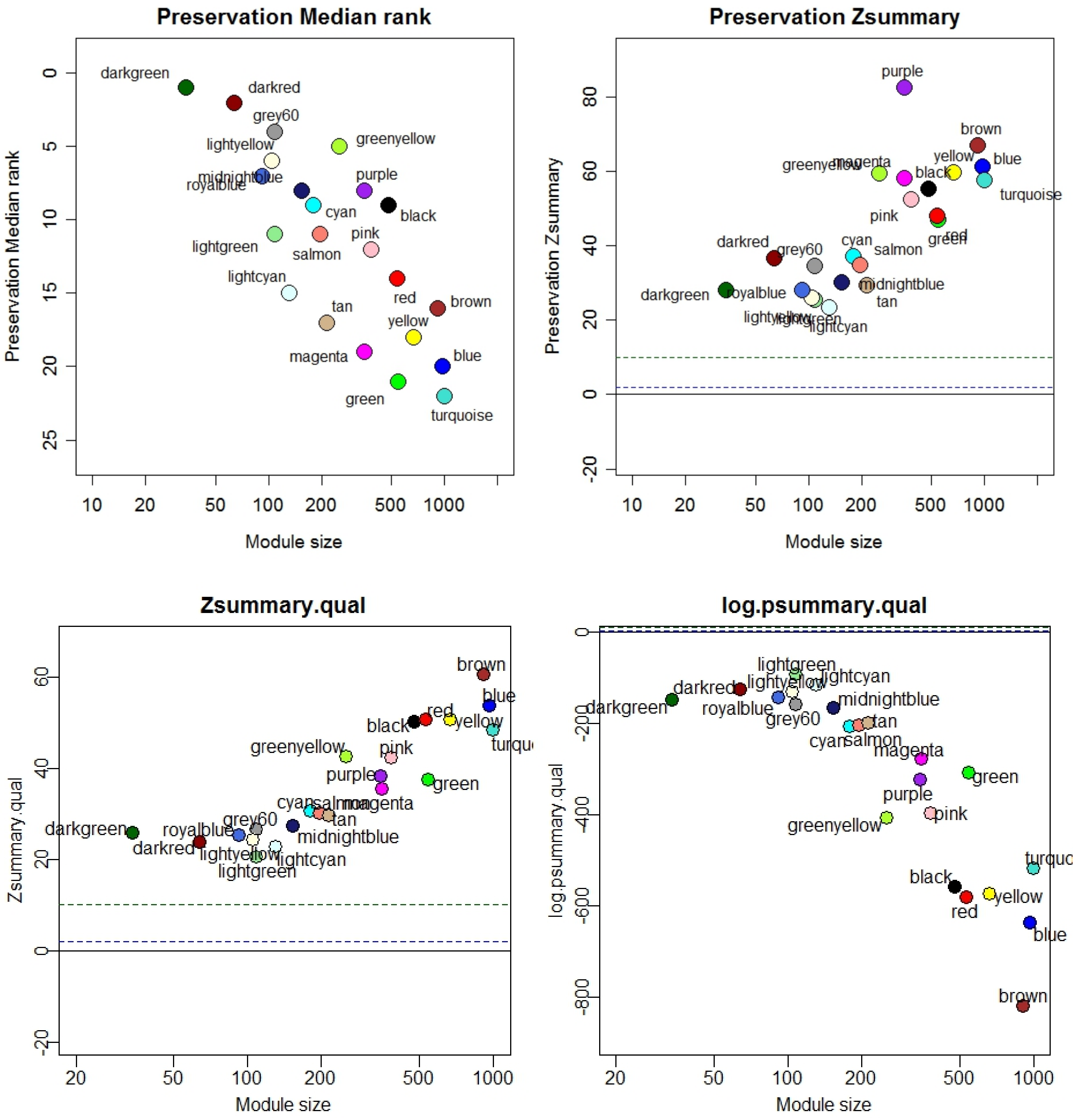

Appendix Figure S2. Module preservation analysis and quality scores. Zsummary statistic (e.g., Zsummary > 10 means highly preserved, Zsummary in between 2 and 10 is weak to moderate preservation, Zsummary<2 is little to no preservation and median rank (modules with lowest rank are highly preserved). Median Rank and Zsummary values indicate high module preservation between the combined ASD + control dataset and itself. Zsummary.qual and related log.p.qual statistics show that module preservation is of high quality and statistically significant against random chance.

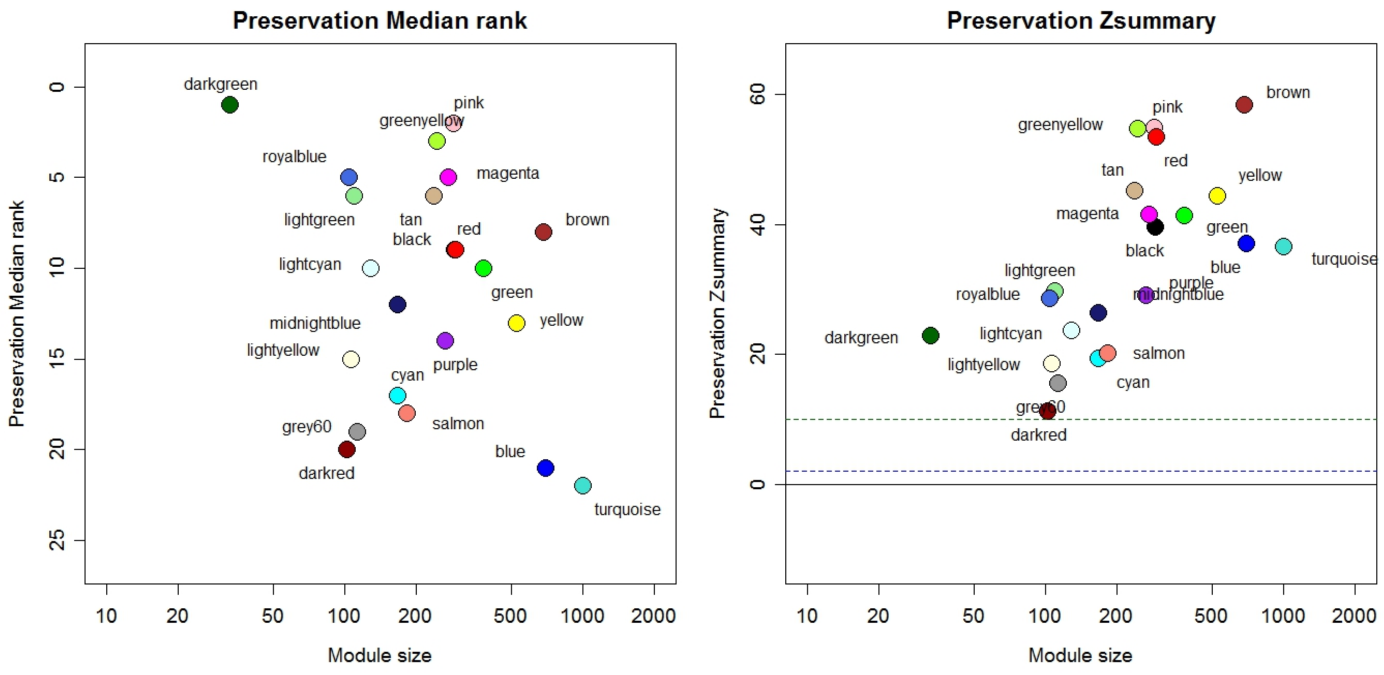

Appendix Figure S3. Module preservation analysis between ASD and control datasets. Zsummary statistic (e.g., Zsummary > 10 means highly preserved, Zsummary in between 2 and 10 is weak to moderate preservation, Zsummary<2 is little to no preservation and median rank (modules with lowest rank are highly preserved). Median Rank and Zsummary values indicate high module preservation between ASD and control datasets.

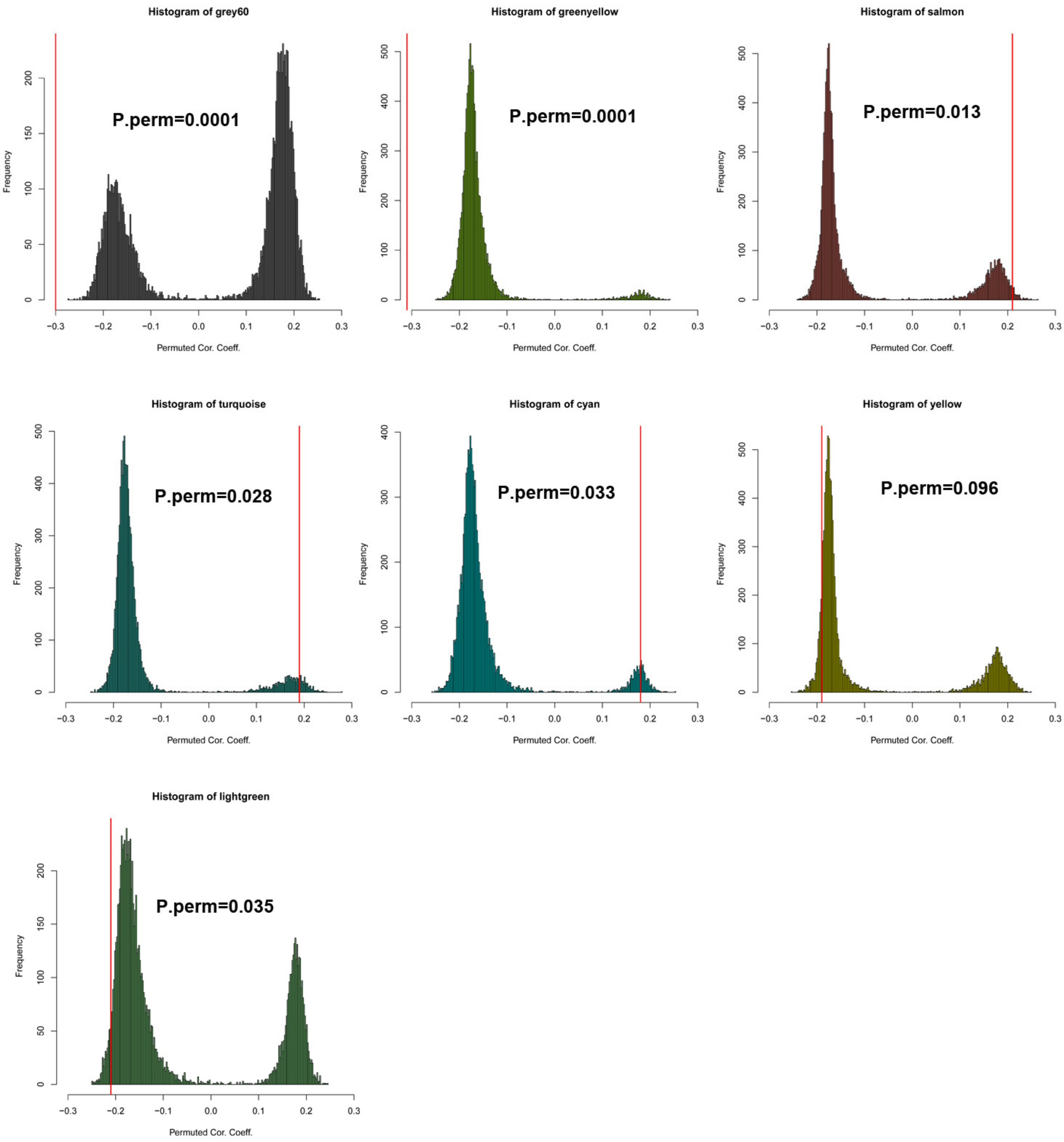

Appendix Figure S4. Permutation analysis of the seven modules associated with TBV measures in the combined WGCNA analysis. Distributions of 10000 correlation tests between randomly generated ME values and TBV measures across all subjects. All modules but yellow displayed significant association with TBV against chance.

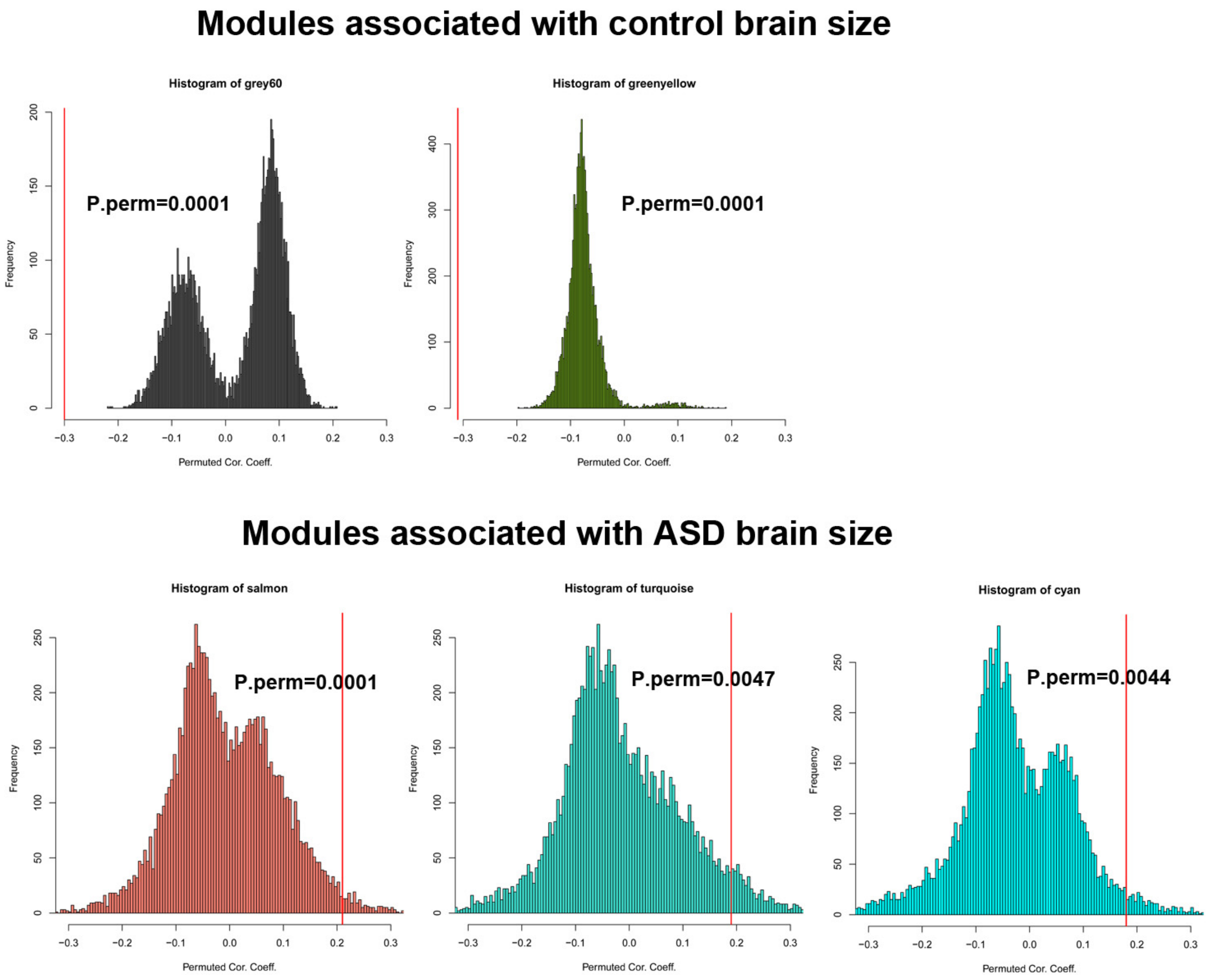

Appendix Figure S5. Permutation analysis of the top modules associated with TBV measures in ASD and Control groups. Distributions of 10000 correlation tests between randomly generated ME values and TBV measures in each group separately. All modules displayed significant association with TBV against chance.

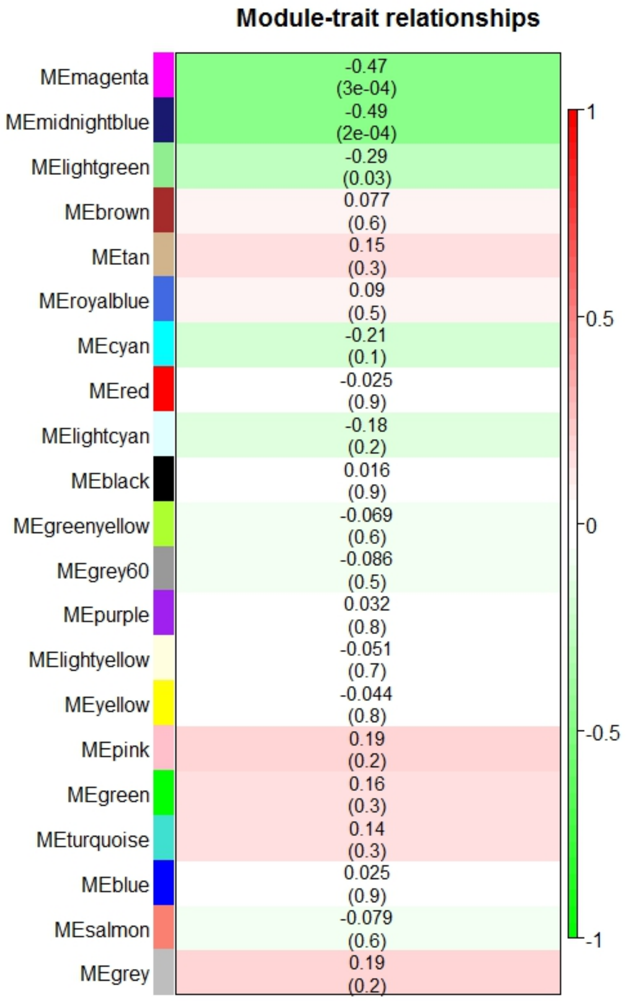

Appendix Figure S6. Association analysis between MEs and TBV measures using WGCNA on control toddlers. Two modules, enriched in cell cycle and protein folding genes were associated with TBV measures in control toddlers (see magenta and midnightblue respectively; see also File E1). Module grey contains genes that were left out from the co-expression analysis.

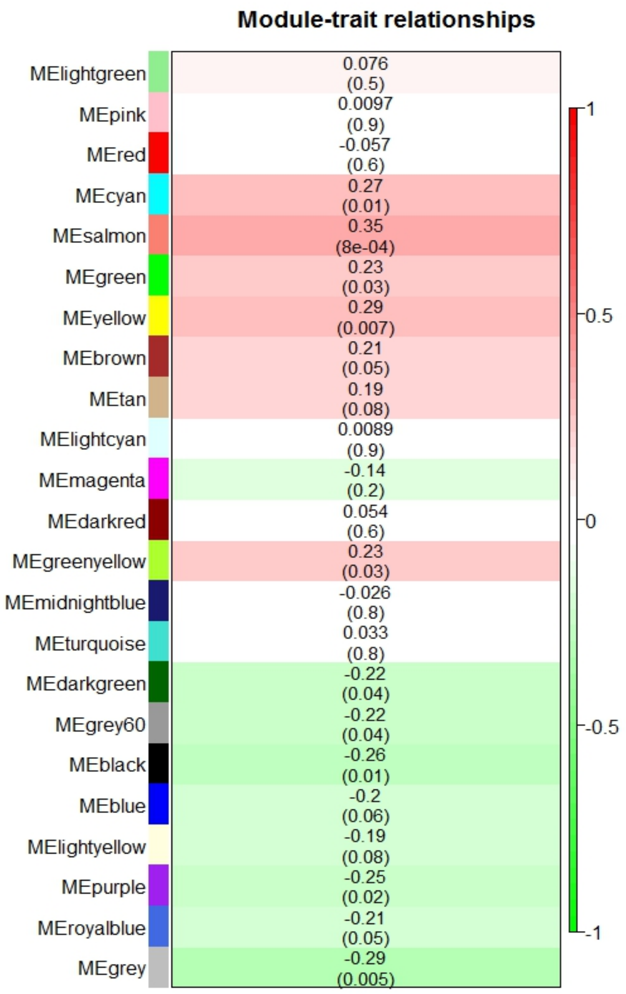

Appendix Figure S7. Association analysis between MEs and TBV measures using WGCNA on ASD toddlers. While nine different modules displayed modest association (r>0.2, *P*<0.05) with TBV in ASD, the strongest association (salmon, r=0.35) was again with the module enriched in cell adhesion genes (see also File E1). Module grey contains genes that were left out from the co-expression analysis.

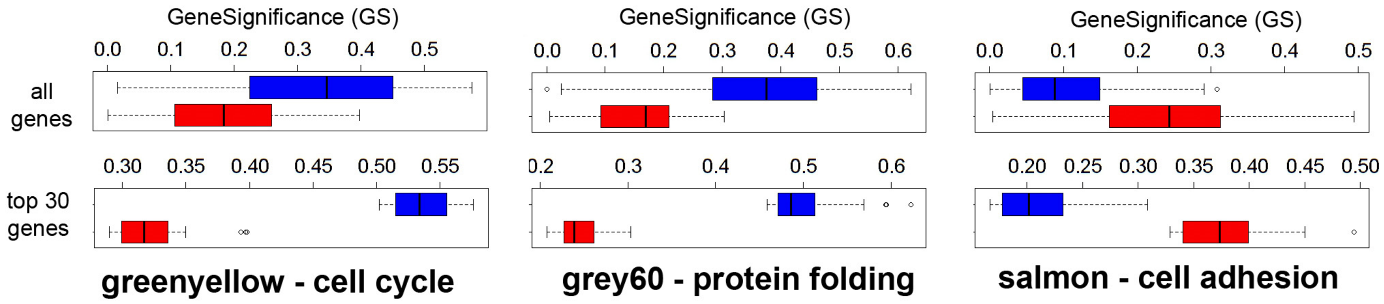

Module	Analysis	t-value	Df	P-value
greenyellow	All genes	14.9177	506	2.2e-16
greenyellow	Top 30 genes	31.3059	58	2.2e-16
grey60	All genes	13.9923	224	2.2e-16
grey60	Top 30 genes	27.8284	58	2.2e-16
salmon	All genes	-14.7129	306	2.2e-16
salmon	Top 30 genes	-15.4298	58	2.2e-16

Appendix Figure S8. Boxplots and T-test analysis of Gene Significance. Gene significance scores display significantly different distributions between ASD (red) and control (blue) toddlers.

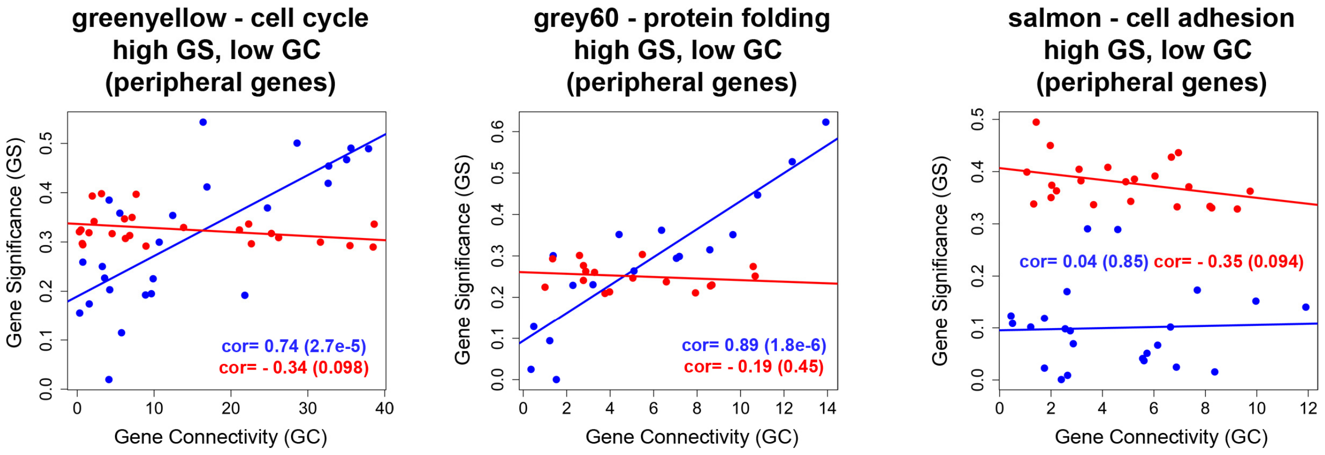

Appendix Figure S9. Linear correlations for peripheral genes with high relevance to ASD brain measures. Scatterplots dislaying correlations between GS and GC in both ASD and control toddlers. The genes selected in these plots are those that had the top 30 highest GS scores in ASD, but which were not within the top 30 in GC scores, and are thus considered ‘peripheral’ in the co-expression networks.

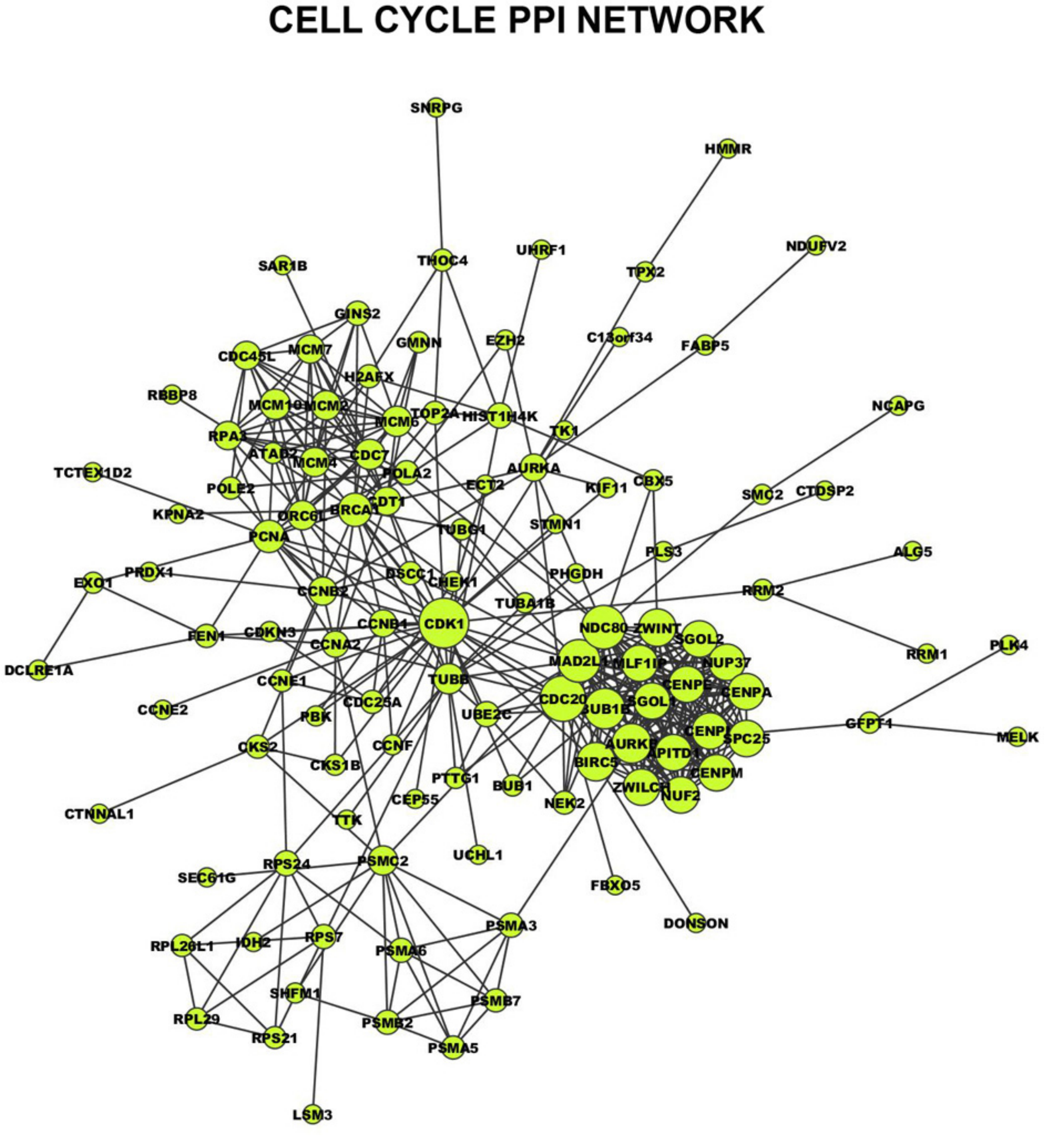

Appendix Figure S10. Topological mapping of cell cycle hub-genes in a protein-protein interaction (PPI) network. A) Reference PPI network for cell cycle genes (GreenYellow module/color). Node (circle) size is proportional to the number of actual biologically driven PPIs (not to the hub-gene connectivity scores based on co-expression used in the top 30 ranking).

APPENDIX TABLES

Appendix Table S1. Module preservation analysis between ASD and control datasets

Module	medianRank.pres	medianRank.qual	Zsummary.pres	Zsummary.qual
brown	8	20	58	44
greenyellow	3	8.5	55	38
pink	2	16.5	55	32
red	9	3.5	54	45
tan	6	6	45	37
yellow	13	16.5	44	43
magenta	5	10.5	42	40
green	10	13	41	44
black	9	10	40	41
blue	21	22	37	44
turquoise	22	18	37	60
lightgreen	6	11.5	30	24
purple	14	6	29	41
royalblue	5	2	29	26
grey	24	24	27	-19
midnightblue	12	15	26	27
lightcyan	10	6	24	27
darkgreen	1	1	23	23
salmon	18	9	20	32
cyan	17	20	19	27
lightyellow	15	20	19	18
gold	23	23	16	1.6
grey60	19	14	16	22
darkred	20	3.5	11	26

Appendix Table S2. Bootstrapped (10,000 resamples) 95% confidence intervals for correlation estimates in TD and ASD

	TD		ASD	
**Module**	**Lower bound**	**Upper bound**	**Lower bound**	**Upper bound**
cyan	-0.2494	0.3006	0.0411	0.4810
greenyellow	-0.6591	-0.2089	-0.4892	0.0437
grey60	-0.6912	-0.2352	-0.4065	0.0161
lightgreen	-0.6125	0.0952	-0.4106	0.1075
salmon	-0.6012	0.0308	-0.2074	0.2798
turquoise	-0.2852	0.4334	0.0713	0.5124
yellow	-0.4689	0.2483	-0.4330	-0.0261

Appendix Table S3. Linear regression analysis with age-correction and ANOVA testing

RESIDUALS CALCULATED FROM CONTROL MEASURES				
**Module**	**Pearson cor**	**Linear model 1**	**Linear model 2**	**Model comparison**
**Control**	**pValue, cor.test(TBV~age)**	**Rsq, lm(TBV~age)**	**Rsq, lm(TBV~ME+age)**	**pValue, anova(lm1, lm2)**
GreenYellow	0.852	0.001	0.258	0.001**
Grey60	0.852	0.001	0.259	0.001**
Salmon	0.852	0.001	0.009	0.593
Cyan	0.852	0.001	0.003	0.797
Turquoise	0.852	0.001	0.005	0.724
Yellow	0.852	0.001	0.018	0.436
**ASD**	**pValue, cor.test(TBV~age)**	**Rsq, lm(TBV~age)**	**Rsq, lm(TBV~ME+age)**	**pValue, anova(lm1, lm2)**
GreenYellow	0.004**	0.124	0.142	0.261
Grey60	0.004**	0.124	0.136	0.357
Salmon	0.004**	0.124	0.217	0.008**
Cyan	0.004**	0.124	0.179	0.046*
Turquoise	0.004**	0.124	0.157	0.122
Yellow	0.004**	0.124	0.153	0.149

Appendix Table S4. Permutation analysis testing between-group difference (TD vs ASD) in correlation strength (10,000 permutations).

Module	rTD	rASD	zDiff_real	pValue
greenyellow	-0.440018973	-0.238972702	-0.228570023	0.177082292
grey60	-0.472288151	-0.204066705	-0.306038998	0.067693231
salmon	-0.080861822	0.348149398	-0.444375118	0.01059894
cyan	0.037118013	0.267513857	-0.237049048	0.116888311
turquoise	0.066477979	0.29185534	-0.23401699	0.142385761
yellow	-0.12174427	-0.237685929	0.119968885	0.277072293
lightgreen	-0.284887484	-0.177027937	-0.114080474	0.318468153

Appendix Table S5. Module preservation analysis between blood and BrainSpan datasets.

moduleColors	medianRank.pres	medianRank.qual	Zsummary.pres	Zsummary.qual
greenyellow	1	2	14	35
brown	6	14.5	12	51
turquoise	13	20	9.8	52
blue	11	18	8.5	49
tan	3	16	7.4	22
red	6	11.5	6.9	39
green	8	20.5	5.2	33
gold	17	23	5.1	0.063
grey	19	24	4.5	-25
pink	8	22	3.1	9.8
salmon	13	6.5	2.6	26
lightgreen	17	6	1.6	19
royalblue	11	5	1.4	22
black	15	10	1.3	36
cyan	16	9	1.3	21
magenta	19	18	0.67	24
yellow	21	13.5	0.5	44
darkred	9	1	0.45	9.5
lightyellow	16	7.5	0.36	16
purple	19	12	0.24	25
grey60	22	3	0.076	20
midnightblue	14	16.5	-0.16	17
lightcyan	18	12	-0.36	19
darkgreen	5	11.5	-0.41	2.4

Appendix Table S6. Permutation analyses testing between-group difference (TD vs ASD) in correlation strength (10,000 permutations)**.**

**Subnetwork**	**Control cor. (pval)**	**ASD cor. (pval)**	**Zdiff**	**Perm. Pval**
CHD8	-0.41(0.009)	-0.075(0.55)	0.3655	0.0353
APH1A	0.21(0.19)	0.24(0.049)	0.0325	0.4413
NR3C2	0.33(0.045)	0.028(0.82)	-0.3112	0.0897

Appendix Table S7. CHD8 subnetwork linear regression analysis with age-correction and ANOVA testing

**CHD8**	**Linear model 1**	**Linear model 2**	**Model comparison**
	**Rsq, lm(TBV~age)**	**Rsq, lm(TBV~ME+age)**	**pValue, anova(lm1, lm2)**
ASD	0.1239	0.1272	0.6282
Control	0.000975	0.2297	0.002737**
