# Supplementary material for: Cell cycle networks link gene expression dysregulation, mutation, and brain maldevelopment in autistic toddlers
Source: Mol Syst Biol. 2015 Dec 14;11(12):841. doi: 10.15252/msb.20156108 (PMC4704485; doi:10.15252/msb.20156108)
Supplement: Supplementary file 2 — Expanded View Figures PDF [file MSB-11-841-s002.pdf]

Expanded View Figures

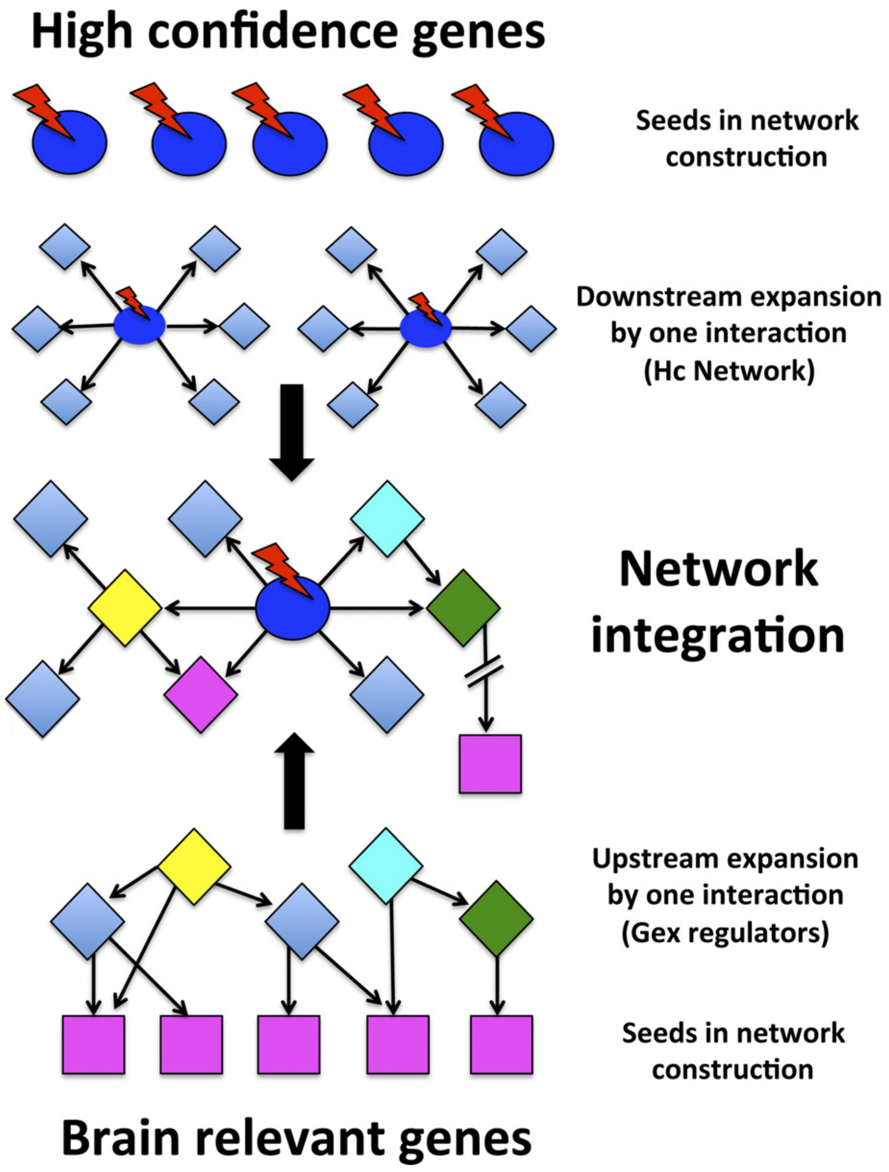

Figure EV1. Schematic of the approach used to generate the High-confidence (Hc) network.
Thirty-two (32) Hc genes (blue circles) were mapped onto Metacore GeneGo to construct a Hc network. Each Hc gene was used as node to identify direct downstream targets (diamond shapes). Red lightning bolts represent the possible presence of mutations affecting the Hc genes. Similarly, twenty-three (23) brain-relevant genes (purple squares) were used in Metacore to look for direct upstream regulatory genes (yellow, cyan, green diamond shapes). Downstream and upstream targets were integrated into a final Hc network.

© 2015 The Authors
